# High-density lipoproteins and COVID-19: preparing the next pandemic

**DOI:** 10.1016/j.jlr.2025.100779

**Published:** 2025-03-14

**Authors:** Marie Laurine Apalama, Floran Begue, Sébastien Tanaka, Maxime Cournot, David Couret, Olivier Meilhac, Mohammad Ryadh Pokeerbux

**Affiliations:** 1 Université de La Réunion, UMR Diabète Athérothrombose Réunion Océan Indien (DéTROI), INSERM U1188, Saint-Pierre, France; 2 USMD, Délégation de la Recherche Clinique et de l'Innovation, CHU de La Réunion, Saint-Pierre, France; 3 AP-HP, Service d'Anesthésie-Réanimation, CHU Bichat-Claude Bernard, Paris, France; 4 Clinique Les Orchidées, Groupe de santé Clinifutur, Le Port, France; 5 Service de Neuroréanimation, CHU de la Réunion, Saint-Pierre, France; 6 INSERM CIC1410, Plateforme de Recherche Clinique et Translationnelle, CHU de La Réunion, Saint-Pierre, France; 7 Service de Médecine Interne et Polyvalente, CHU de la Réunion, Saint-Pierre, France

**Keywords:** lipids, epidemiology, drug therapy, inflammation, dyslipidemia, high-density lipoproteins, COVID-19, reconstituted HDLs, proteomics, lipidomics

## Abstract

High-density lipoproteins (HDLs) are heterogeneous particles with pleiotropic functions including anti-inflammatory and anti-infectious effects. In clinical studies, lower HDL-associated cholesterol (HDL-C) concentration has been associated with severe acute respiratory syndrome coronavirus 2 (SARS-CoV-2) infection, severity, and mortality. A reduction in the number of HDL particles, particularly small ones has been observed with alterations in their protein and lipid composition impairing their functions. These observations have supported HDL supplementation with promising results in small preliminary studies. This review summarizes available evidence to better understand the two-way interaction between HDLs and Coronavirus disease 2019 (COVID-19) and guide future HDL-based therapies for preparing for the next pandemic.

High-density lipoproteins (HDLs) are heterogeneous particles characterized by their density and size that consist of lipids (including phospholipids, cholesterol, sphingolipids, and triglycerides) and proteins, mainly apolipoprotein A-1 (APOA1) (1, 2, 3). HDLs are responsible for reverse cholesterol transport from peripheral tissues to the liver and exert other pleiotropic functions such as anti-inflammatory and anti-infectious effects (4). For example, HDLs exert their anti-inflammatory effects by inhibiting the expression of leukocyte adhesion molecules on endothelial cells and by reducing the secretion of pro-inflammatory cytokines by monocytes. HDLs also limit neutrophil activation and degranulation under inflammatory stress conditions and inhibit damage associated with the proteolytic activity of elastase (5, 6, 7, 8, 9, 10, 11). I Importantly for their anti-infectious effects, compared to other lipoproteins, HDLs show the highest binding capacity to lipopolysaccharide (LPS), which is the major component of the outer membrane of Gram-negative bacteria, and to lipoteichoic acids (LTA) on Gram-positive bacteria membrane (12, 13, 14). This ability to bind to bacterial endotoxins also enables them to inhibit their inflammatory effects and allow their elimination via the hepato-biliary route. In routine assays, only the cholesterol contained in the HDL fraction is measured, a molecule that does not carry the protective effects of these lipoproteins and does not reflect their functionality. It is therefore HDL-C (HDL-associated cholesterol concentration) that is monitored in most clinical studies. Low HDL-C is well documented in septic patients and is associated with severity, multi-organ dysfunction, and mortality (15, 16, 17, 18). Although variations in the lipid profile during viral disease are less extensively studied, there is evidence from studies of virus infections, such as with human immunodeficiency virus (HIV) and dengue virus, that these infections are associated with low cholesterol levels, including a reduction in HDL-C as observed during bacterial sepsis (19, 20).

In this review of the literature, we will present the clinical studies concerning HDL-C variations in Coronavirus disease 2019 (COVID-19), as well as the alterations in the size and composition of these lipoproteins in the course of this pathology. Besides cholesterol, many proteins and lipids associated with HDL particles vary in inflammatory and acute infection conditions. We will also discuss the impact of COVID-19 on lipid profiles and possible consequences for the incidence of cardiometabolic diseases. Finally, the therapeutic potential of reconstituted HDLs (rHDLs) to treat viral as well as bacterial infections will be considered based on their pleiotropic functions, with a particular focus on intervention studies already published or underway.

## Clinical Evidence of HDL-C/COVID-19 Interplay

Early reports following the COVID-19 outbreak have shown that obesity and existing cardiovascular risk factors were associated with the risk of infection with severe acute respiratory syndrome coronavirus 2 (SARS-CoV-2), hospital admission, severity, and mortality (21, 22, 23, 24). Subsequently, profound changes in the lipid profile of patients in the acute phase of COVID-19 compared to healthy individuals have also been reported, including a fall in low-density lipoprotein cholesterol (LDL-C) and total cholesterol, as well as a decrease in HDL-C in critical cases (25). A recent meta-analysis confirmed that dyslipidemia is associated with severity and mortality of COVID-19 (26). Table 1 shows the main studies reporting HDL-C level changes in COVID-19. In general, these studies are observational in nature, and while they provide valuable insight into the interaction between HDL-C and COVID-19, they cannot establish causality due to the potential for various biases such as confounding bias and reverse causality.

**Table 1:** Main studies investigating HDL-C levels in COVID-19 patients

Timeframe	Author	Year	Country	Type of Study	Population	N	Main results
Pre-pandemic HDL-C	Chidambaram (27)	2022	USA	Retrospective	Individuals > 18 years positive for COVID-19	1,340	Higher antecedent HDL-C, but not LDL-C, TC, or TG, levels were associated with a lower SARS-CoV-2 infection risk (RR 0.63, 95% CI 0.46-0.86).
	Lassale (28)	2021	UK	Prospective cohort study	UK biobank	1845	Higher pre-pandemic HDL-C associated with lower risk of hospitalization, a 0.2 mmol/L increase in HDL-C was associated with a 7% lower risk (OR 0.93, 95% CI 0.90-0.96).
HDL-C during acute phase	Aparisi (29)	2021	Spain	Retrospective	Consecutively admitted patients	654	Lower HDL-C before admission (47.2 vs. 52.6 mg/dl; *P* = 0.004) and 7th day (27 vs. 34 mg/dl; *P* = 0.011) positively associated with 30-days mortality.
	Begue (30)	2021	France	Prospective	ICU patients	8	Lower HDL-C 0.77 mmol/l (IQR 0.49–0.83) compared to control subjects.
	Ding (31)	2020	China	Retrospective	Patients with SARS-CoV-2 nucleic acid positive time exceeding 14 days	115	HDL-C reduction as an independent risk factor of viral clearance time (OR 0.53, 95% CI 0.31-0.91, *P* = 0.021).
	Hu (32)	2020	China	Retrospective	Hospitalized patients	114	Decreased HDL-C compared to healthy controls (1.01 vs. 1.21 mmol/L, *P* < 0.001) and associated with severity (OR 0.023, 95% CI 0.002-0.227). HDL-C negatively correlated with CRP and positively correlated with lymphocytes count.
	Huang (33)	2021	China	Cross-sectional retrospective study	Hospitalized patients	218	Lower HDL-C in COVID-19 patients compared to healthy controls (1.02 ± 0.28 vs. 1.52 ± 0.55 mmol/L) and in severe patients compared to non-severe ones (0.83 ± 1.67 vs. 1.15 ± 0.27 mmol/L).
	Lv (34)	2021	China	Retrospective	Hospitalized patients	94	Lower HDL-C in COVID-19 patients compared to healthy controls (0.88 vs. 1.35 mmol/l, *P* < 0.001). No association of HDL-C with severity.
	Masana (35)	2021	Spain	Retrospective	Patients with lipid profile before and during hospitalization	1,305	Lower HDL-C in severe patients compared to mild ones (0.73 mmol/L vs. 0.88 mmol/L, *P* < 0.001). Severe outcome was associated with lower HDL-C.
	Ouyang (36)	2020	China	Retrospective	Hospitalized patients	107	Gradual increase in HDL-C in survivors compared to non-survivors.
	Qin (37)	2020	China	Retrospective	Hospitalized patients	248	HDL-C higher in severe cases compares to common cases (1.03 vs. 0.86 mmol/L, *P* < 0.001). HDL-C lower in patients with LOS>29 days (1.10 vs. 1.29 mmol/L, *P* < 0.05). Gradual increase in HDL-C during hospitalization.
	Sun (38)	2020	China	Prospective	Severe and critically ill patients	99	Lower HDL-C associated with severe disease (OR 0.64, 95% CI 0.46-0.91, *P* = 0.01) and higher mortality. HDL-C negatively correlated with CRP and IL-6.
	Tanaka (39)	2020	France	Prospective	ICU patients	48	Low HDL-C at admission (0.7 IQR 0.5–0.9 mmol/L). Statistically significant increase in HDL-C during the ICU stay. No relationship between HDL-C and mortality on day 28 (log-rank *P* = 0.554)
	Turgay (40)	2021	Turkey	Retrospective	Hospitalized patients	139	Lower HDL-C in deceased patients (28.5 vs. 44.0 mg/dl, *P* < 0.001).
	Wang (41)	2020	China	Retrospective	Hospitalized patients	228	Lower HDL-C in COVID-19 patients compared to controls, and associated with higher risk of developing severe events (HR 2.83, 95%IC 1.19–6.71, *P* = 0.019).
	Wang (42)	2020	China	Cross-sectional study	Hospitalized patients	143	Lower HDL-C associated with disease severity (r = − 0.362, *P* < 0.001).
	Wei (25)	2020	China	Retrospective	Hospitalized patients	597	Lower HDL-C compared to control. Lower HDL-C in critical cases compared to mild and severe cases (36 vs. 50 mg/dl, *P* < 0.05). CRP levels inversely correlated with HDL-C (r = −0.351, *P* < 0.001).
	Zhang (43)	2020	China	Retrospective	Hospitalized patients with type 2 diabetes	74	Lower HDL-C in severe patients (0.92 vs. 1.08 mmol/L, *P* = 0.02).
	Zhang (44)	2020	China	Restrospective	Severe or critically ill patients	98	Lower HDL-C in critically ill patients (0.8 vs. 1.0 mmol/L, *P* = 0.001) and in non-survivors (0.7 vs. 0.9 mmol/L, *P* = 0.002).
HDL-C during post-acute phase	Al-zadjali (45)	2024	Oman	Prospective	Long-COVID-19 individuals affected by the original alpha strain and unvaccinated	88	HDL-C lower in severe group compared to mild/moderate group (1.1 vs. 1.3 mmol/L). HDL-C in severe patients lower up to four months after infection.
	Deuel (46)	2022	Switzerland	Longitudinal cohort study	Unvaccinated, young adults of the Swiss ArmedForces	501	Increased total cholesterol and LDL-C levels, with no differences in HDL-C between long-COVID-19 participants and controls at 180 days after infection.
	Li (47)	2021	China	3–6 months follow-up study	Follow up of hospitalized recovered patients	107	Increase in HDL-C at follow-up compared to admission in severe/critical cases (55.3 vs. 50.3 mg/dl, *P* = 0.042).
	Xu (48)	2023	US	Cohort study	Patients with positive COVID-19 test and who survived 30 first days of infection, free of dyslipidemia (National health-care databases of the US Department of Veterans Affairs)	51,919	Increased risks and 1-year burdens of incident dyslipidemia (including low HDL-C) and incident lipid lowering medications use in the post-acute phase of COVID-19 infection (HR 1.20, 95% CI 1.16-1.25; burden 15.58, 95% CI 12.52-18.73 per 1,000 people at one year).

CI, confidence interval; HDL-C, high-density lipoprotein cholesterol; HR, hazard ratio; ICU, intensive care unit; LDL-C, low-density lipoprotein cholesterol; LOS, length of stay; N, number of patients with COVID-19 in study; OR, odds ratio; r, correlation coefficient; RR, relative risk; RT-PCR, Reverse transcription polymerase chain reaction; TC, total cholesterol; TG, triglycerides.

### Pre-pandemic HDL-C

Data from the UK biobank have shown that higher pre-pandemic HDL-C levels (sample collected between 2006 and 2010) were significantly associated with a lower risk of subsequent COVID-19 hospitalization. A 0.2 mmol/L increase in HDL-C was associated with a 7% lower risk of hospitalization (odds ratio (OR) 0.93, 95% confidence interval (CI) 0.90–0.96) (28). However, there was no evidence of association of genetically elevated HDL-C levels with SARS-CoV-2 infection (49). Similarly, higher antecedent HDL-C, but not LDL-C nor triglycerides levels were associated with decreased risk of COVID-19 infection in a large US retrospective study. In this study, the risk of SARS-CoV-2 infection among the trajectories of lipid levels during the 2 years antecedent to COVID-19 testing was assessed. The highest trajectory for antecedent serum HDL-C was associated with the lowest SARS-CoV-2 infection risk (risk ratio (RR) 0.63, 95% CI 0.46-0.86) (27). Lower baseline HDL-C levels measured in the last 18 months before COVID-19 have also been reported to be positively associated with 30-day mortality (29).

### HDL-C during acute phase of COVID-19

At hospital admission, patients with SARS-CoV-2 infection showed hypolipidemia with lower HDL-C levels compared to control subjects. Moreover, lower HDL-C levels were associated with the severity of symptoms and predictive of developing a severe form (25, 32, 33, 34, 35, 38, 41, 43, 50). For example, in a retrospective study of 228 adults with COVID-19, after adjusting for age, gender, and underlying diseases, patients with low HDL-C had a higher risk of developing severe cases than those with high HDL-C (hazard ratio (HR) 2.83, 95% CI 1.19-6.71, *P* = 0.02) (50). HDL-C levels were inversely associated with inflammatory biomarkers such as CRP and IL-6 during the infection (32, 38). Lower HDL-C levels have also been associated with mortality of COVID-19 patients (29, 38, 40, 44), including in the case of bacterial superinfection during ICU hospitalization (51). A recent meta-analysis confirmed that HDL-C was lower in patients with COVID-19 than in healthy controls, lower in severe patients, and lower in deceased patients (52).

HDL-C reduction was an independent risk factor of viral clearance time (OR 0.53, 95% CI 0.31-0.91, *P* = 0.02) (31). Compared to deceased patients, survivors have shown a gradual increase in HDL-C during hospitalization (36, 37). Follow-up data at 3–6 months has shown an increase in HDL-C compared to admission in severe patients (47).

Statins are primarily effective in lowering LDL-C levels while they have very minor effects on HDL-C (53). Additionally, they possess pleiotropic effects, including anti-inflammatory, immunomodulatory, and antithrombotic properties that could help treat COVID-19 (54, 55, 56). A large retrospective study found that statin use was associated with a lower risk of mortality in COVID-19 patients, although as an observational study, the effectiveness of statins could be overestimated due to healthy user bias such as healthier behavior and better adherence to other medications (57). Although several individual clinical trials did not show a clear beneficial effect of initiation of statins on clinical deterioration or mortality, a recent meta-analysis found that adjunctive statin therapy significantly reduced case-fatality rate in COVID-19 (RR 0.88, 95% CI 0.80-0.98, I2 = 0%) (57, 58, 59, 60).

### HDL-C during the post-acute phase of COVID-19

In the post-acute phase of COVID-19, patients are at higher risk of incident cardiovascular disease including cerebrovascular disorders, ischemic heart disease, inflammatory heart disease, dysrhythmias, and thrombotic disorders (61). Furthermore, in the post-acute phase, patients show an increased risk of developing dyslipidemia with higher incident use of lipid-lowering medications (HR 1.20, 95% CI 1.16-1.25) (46, 48), and present lower HDL-C, associated with severity of the acute phase of COVID-19 infection (45, 48).

These studies suggest that COVID-19 can have a significant impact on lipid profile, particularly by lowering HDL-C levels, but also that baseline lipid profile before infection is associated with disease severity. Moreover, in the long term, patients are at higher risk of developing dyslipidemia and incident cardiovascular disease. This is therefore a two-way interaction between lipoproteins and COVID-19.

## Structure and Function of HDLs

HDLs are composed of a heterogeneous population of particles that exhibit variation in size, protein, and lipid composition (62). This structural and functional diversity is a crucial factor in their ability to protect against cardiovascular and infectious diseases.

As presented, several studies have reported a reduction in circulating HDL-C levels in patients diagnosed with COVID-19. Furthermore, structural alterations in HDL particles have been observed in patients infected with SARS-CoV-2, in addition to the decrease in circulating levels.

### HDL particle size

In addition to the observed decrease in circulating HDL-C concentrations, a reduction in the number of circulating HDLs was also reported in these patients. In the NIH Lipo-COVID study, serum concentrations of HDLs were found to be 40% below reference values (63). In particular, several studies have demonstrated a decline in the number of small HDLs in individuals diagnosed with SARS-CoV-2 infection (64, 65). These small HDLs, which are the most functional (66), play a crucial role in cholesterol efflux via the ABCA1 transporter. A negative correlation was observed between the cholesterol efflux capacity of HDLs and disease severity; although this property of HDLs is not directly involved in their anti-inflammatory and anti-infectious effects, this alteration could reflect dysfunction of these lipoproteins (64). The reduction in small HDLs and their cholesterol efflux capacity is particularly pronounced in severe forms of the disease. These observations suggest that the determination of HDL number and size may potentially serve as biomarkers for assessing the severity of infection (64).

### HDL proteome

Apolipoproteins, the primary constituents of lipoproteins, are instrumental in defining their functional characteristics and metabolic functions. The plasma concentrations of most apolipoproteins are markedly diminished in patients diagnosed with COVID-19, as well as lecithin cholesterol acyltransferase (LCAT) lipase, an enzyme indispensable for HDL maturation (67, 68). These alterations may reflect perturbations in hepatic HDL biogenesis and/or increased particle clearance, providing an explanation for the observed decrease in HDL levels (67, 69). Global analysis of plasma apolipoprotein concentrations is important but must be complemented by a more detailed study of proteome variations on isolated HDLs to elucidate structural and functional alterations of these particles in response to SARS-CoV-2 infection.

Analysis of the protein and lipid components of HDLs represents a promising avenue of research for elucidating the alterations in lipid metabolism that occur during a SARS-CoV-2 infection. Several studies demonstrated that HDLs in patients infected with SARS-CoV-2 exhibit alterations in their protein composition. In the context of our study, ultracentrifugation was employed to isolate the HDL fraction, and the proteome was analyzed with mass spectrometry. The resulting analysis revealed a significant alteration in the proteome evidenced by a reduction in apolipoproteins, particularly APOA1 and APOM, as well as antioxidant enzymes such as paraoxonase 1 (PON1). Furthermore, enrichment in acute-phase proteins, including SAA-1, SAA-2, AAT, and AGP1, was observed (30). In the early phases of SARS-CoV-2 infection, there is an increase in the concentration of SAA in HDLs (70). Moreover, this protein alteration is correlated with disease severity, as evidenced by the enrichment in SAA-1 paralleled by the depletion in APOM in HDLs of hospitalized patients. A negative correlation has been established between the concentration of APOM and mortality in patients with SARS-CoV-2 infection (71). Conversely, a positive correlation has been identified between SAA-1 concentration and mortality (71). It would be important to evaluate the concentrations of APOM and SAA-1 in other infectious pathologies to substantiate their potential as prognostic indicators. Following remission, the normalization of apolipoprotein abundance was observed, potentially indicating partial recovery of HDL function (71). A recent study has described significant alterations in the HDL proteome involving hemoglobin, cytoskeletal proteins, and amyloid precursor protein of patients with post-COVID-19 syndrome compared to asymptomatic subjects and has suggested that treatment with statins and angiotensin II type 1 receptor blockers modifies the HDL proteome which in turn reduces the inflammatory response of endothelial cells (72).

### HDL lipidome

Lipids, the other major components of HDLs, are also altered during COVID-19. In particular, the lipid core of HDLs is enriched in triglycerides in patients infected with SARS-CoV-2 (73). In contrast, membrane lipids such as free cholesterol and phospholipids are decreased in these patients. These lipid changes can discriminate SARS-CoV-2-infected individuals from healthy individuals (73, 74).

In addition, HDLs carry a sphingolipid, sphingosine-1-phosphate (S1P), which has endothelial protective properties. The concentration of S1P is reduced in COVID-19 patients, probably due to its correlation with the presence of APOM, whose levels are reduced in the HDLs of COVID-19 patients (30, 75). S1P is also considered a prognostic marker for the severity of COVID-19, particularly with regard to ICU admission (75, 76). These changes in HDL lipid composition highlight its potential as a biomarker in the context of infectious diseases such as COVID-19. Furthermore, sphingosine-1-phosphate receptor ligands (SRLs) may reduce lung damage in viral pneumonia, and ozanimod, a novel sphingosine-1-phosphate receptor ligand, has been recently studied in COVID-19 patients in a randomized open-label pilot trial (77). Compared to the standard of care, patients treated with ozanimod showed non-significant reductions in the duration of respiratory support, duration of hospitalization and median time to clinical improvement. To our knowledge, reconstituted HDL particles enriched in S1P have not yet been explored as a therapeutic option in COVID-19.

### HDL function

In addition to the structural and composition changes in HDLs observed during COVID-19, the functional properties of these lipoproteins are also altered. Indeed, the main function of HDLs, cholesterol efflux capacity, is impaired in patients infected with SARS-CoV-2. A significant decrease in cholesterol efflux activity by HDL has been reported in COVID-19 patients, and a negative correlation between cholesterol efflux and disease severity has been observed (74). In addition, acute-phase SAA levels, SAA-1 and SAA-2 isoforms, increase up to 15-fold in patients requiring hospitalization compared to those with mild symptoms (71). This increase in SAA-1 and SAA-2, often observed in acute systemic inflammatory conditions such as COVID-19, contributes to the disruption of HDL-mediated cholesterol efflux due to protein modifications of these lipoproteins (78, 79).

In addition to impaired cholesterol efflux capacity, HDLs from COVID-19 patients show a marked loss of protective functions. Studies have highlighted an impairment of HDL antioxidant functions, in particular a decrease in PON1-mediated arylesterase activity, as well as a reduction in their anti-inflammatory and antioxidant properties (30, 69). Furthermore, a decrease in LCAT activity and serum concentrations of this enzyme was observed in COVID-19 patients, which may explain the reduction in circulating HDL-C concentrations (67).

From the point of view of HDL composition, as determined by proteomics and lipidomics, the differences between COVID-19 and bacterial sepsis appear to be relatively minor compared to healthy subjects (30, 74). Even in the presence of sterile inflammation, there are certain common features that render HDLs dysfunctional, such as the accumulation of SAA, suggesting that severe systemic inflammation is the major determinant of qualitative and quantitative changes in HDLs (51). In the case of SARS-CoV-2 infections, very few studies have demonstrated a direct interaction of viral elements with HDL particles which would suggest an improvement in pathogen clearance as observed in bacterial sepsis (25, 80).

The functional and enzymatic changes associated with the severity of COVID-19 strengthen the use of HDLs as potential biomarkers of infection severity and possibly as a therapeutic target. Indeed, high HDL-C concentrations are associated with a lower risk of developing COVID-19 and severe forms of COVID-19, suggesting that supplementation with functional HDLs may represent a therapeutic option (27, 81).

## HDL-Based Therapies

### HDL in the treatment of bacterial infection

Reconstituted HDLs have demonstrated efficacy in treating bacterial sepsis. In the murine model, CSL-111, a commercial rHDL product, significantly reduced bacterial presence and improved survival rates following infections with E. coli or Pseudomonas aeruginosa. Additionally, a reduction in inflammation markers in plasma and organs was observed. Furthermore, a decrease in bacterial burden was noted, highlighting the protective role of HDLs in organ function and clearance of bacterial lipopolysaccharides (82). In a translational study, CER-001, another rHDL, was administered to 20 patients with sepsis. The Phase 2a pilot study was conducted to compare conventional treatment of sepsis with short-term treatment with CER-001. The results showed a significant reduction in the concentration of LPS in the blood compared to patients receiving conventional treatment. In addition, administration of CER-001 was associated with improved patient outcomes, including a reduced risk of developing severe acute kidney injury. Thus, CER-001 appears to be a promising therapeutic strategy against sepsis and its complications (83).

### HDL and viral infection

Viral infections, particularly SARS-CoV-2, have been shown to have a marked impact on lipid metabolism in infected patients. A correlation has been demonstrated between a reduction in HDL-C and disease severity, as well as changes in protein composition (30). Based on the decrease of HDL-C concentration and the loss of HDL function, the exploration of rHDLs as a potential therapeutic strategy against viral infections, in particular SARS-CoV-2, is an important path to explore. However, some studies may suggest that HDLs facilitate the entry of SARS-CoV-2 into cells, suggesting that HDL supplementation could be deleterious in the context of COVID-19. The study by Wei and colleagues shows that the scavenger receptor SR-B1 (receptor for HDLs) promotes viral entry by enhancing the attachment of the spike protein to the ACE2 receptor. In vitro experiments have shown that the S1 subunit of the SARS-CoV-2 spike protein can bind to cholesterol in HDL, enabling virus internalization. Inhibition of SR-B1 by antagonists of this receptor, or the use of antibodies that block recognition of cholesterol present on HDL, reduces viral infection (25). This study was challenged by Cho and colleagues, showing that the commercial HDLs used in this work were not of adequate quality, containing notably high levels of apolipoprotein A2 (80). Given that the composition of HDLs is crucial to their functions, including their anti-viral effects (84), Wei's study should be treated with caution.

A preliminary study suggested that CER-001 can improve inflammatory profile. A patient with severe COVID-19 admitted to the intensive care unit with pulmonary dysfunction characterized by acute respiratory distress syndrome received CER-001 intravenous infusions, every 12 h for 3 consecutive days. She suffered an intense inflammatory state at ICU admission with very high levels of pro-inflammatory cytokines and an altered lipid profile with markedly reduced HDL-C levels. CER-001 infusions improved this patient's inflammatory profile by reducing cytokine levels and also improved her clinical and respiratory status (65). A subsequent clinical trial investigated the protective effects of CER-001 in addition to standard of care in four patients infected with COVID-19. Results showed an increase in both APOA1 and HDL-C levels. Three of the patients showed rapid clinical improvement with reduced oxygen requirements and decreased inflammatory markers (85). Altogether, these preliminary results suggest that rHDLs may help modulate the inflammatory response associated with SARS-CoV-2 infection.

### Future of HDL therapeutics

**Fig. 1:**
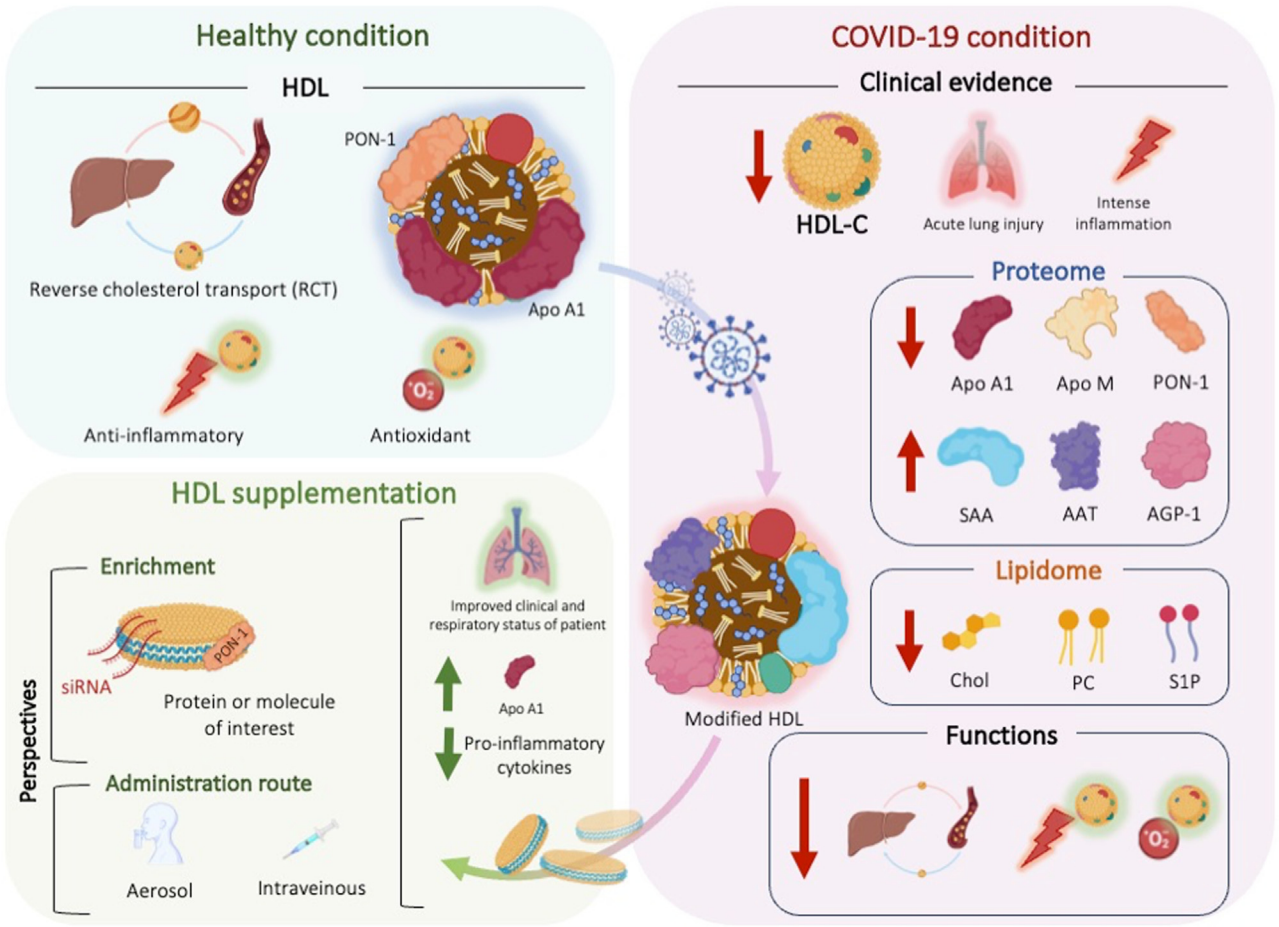
Structural and functional changes in HDL under COVID-19 conditions and HDL mimetic supplementation. Under normal conditions, HDL (high-density lipoprotein) not only plays a role in cholesterol transport but also possesses anti-inflammatory and antioxidant properties. However, following infection with SARS-CoV-2, there is a noticeable decline in HDL levels, along with significant structural alterations. Key proteins such as apo A1 and apo M are reduced, while proinflammatory proteins such as SAA increase. Additionally, the lipid composition of HDL is affected, with decreases in cholesterol and phospholipids. These changes impair HDL's functionality. HDL mimetic supplementation has shown promising results in clinical cases, highlighting the importance of optimizing their composition and delivery methods to better target specific diseases.

These initial results appear promising for the treatment of COVID-19. Other viral infections, such as dengue fever, also exhibit lipid and inflammatory profiles similar to those of COVID-19. Consequently, treatment with rHDLs could also be considered for this type of infection. In a dengue virus (DENV) infection model, nonstructural protein 1 (NS1)-treated cells show enrichment of lipid rafts on the surface which facilitates DENV attachment to the cell membrane. APOA1 addition inhibits NS1-induced cell activation and lipid raft accumulation, thereby inhibiting the NS1 facilitation effect on DENV attachment to the cell membrane (86). An infusion of rHDLs could therefore be considered in the acute phase of viral or bacterial infections, which share the common feature of massive inflammation associated with changes in lipid profiles, with a drop in total cholesterol, LDL-C, and above all HDL-C, as well as an increase in triglycerides. The HDLs currently used are composed of APOA1 and phospholipids; however, the protective properties of HDLs also rely on other proteins or lipids they carry. The potential use of rHDLs as vectors for therapeutic molecules could be considered, allowing them to be delivered directly to an inflammatory site. For example, alpha-antitrypsin vectorized by HDLs improves bioavailability in patients with pulmonary emphysema compared with normal treatment (87). Other strategies using siRNA as a novel therapy have also demonstrated in vitro and in vivo efficacy in the treatment of cancers (88, 89). It would therefore be interesting to explore therapies using rHDLs enriched with siRNA, miRNA, or even antisense oligonucleotides to treat viral infections. Figure 1 illustrates the structural and functional changes in HDL under COVID-19 conditions, which can be modulated by rHDL supplementation.

## Conclusion

The functions of HDLs extend beyond reverse cholesterol transport. HDLs, composed of lipids and proteins, have beneficial properties during bacterial or viral infections. Unlike in sepsis due to Gram-negative bacteria, the protective effect of HDLs in viral infections cannot be based on the binding of endotoxins. Rather, HDLs may exert their effects through modulation of systemic inflammation and endothelial protection, the cell type that is at the forefront of the passage of SARS-CoV-2 from the respiratory tract to the bloodstream. Moreover, HDLs under normal conditions also exert major antioxidant effects which are impaired during COVID-19. However, the direct effects of HDLs or APOA1 on SARS-CoV-2 infection are unclear, with contradictory results. Overall, HDLs could be beneficial in COVID-19 through a combination of direct and indirect mechanisms modulating the response to infection. The reduction of HDLs and alterations in their structure during acute inflammation can impair their function, potentially limiting these beneficial effects, hence supporting HDL supplementation. The lessons learned from observational and interventional studies during the COVID-19 pandemic should serve as a foundation for addressing future viral infections. Further investigation of the lipidome and proteome of patients following the acute phase of COVID-19 should also be undertaken to better understand the long-term effects of viral infection on lipid profiles and cardiometabolic complications. Future HDL therapies should be based on precision medicine, which includes determining the optimal mode and timing of administration for specific infections. For instance, aerosol therapy supplementation may be more appropriate for airborne viral infections, while intravenous administration might be more effective for bloodborne viruses. Additionally, tools such as siRNA, miRNA, and specific protein targeting of the pathology should be explored.

## Data availability

All representative data are contained within the article.

## Conflict of interest

The authors state that they have no conflicts of interest with the contents of the article.

## References

[1] Scherer M., Böttcher A., Liebisch G. (2011). Lipid profiling of lipoproteins by electrospray ionization tandem mass spectrometry. Biochim. Biophys. Acta.

[2] Wiesner P., Leidl K., Boettcher A., Schmitz G., Liebisch G. (2009). Lipid profiling of FPLC-separated lipoprotein fractions by electrospray ionization tandem mass spectrometry. J. Lipid Res..

[3] Karlsson H., Leanderson P., Tagesson C., Lindahl M. (2005). Lipoproteomics II: mapping of proteins in high-density lipoprotein using two-dimensional gel electrophoresis and mass spectrometry. Proteomics.

[4] Tran-Dinh A., Diallo D., Delbosc S., Varela-Perez L.M., Dang Q., Lapergue B. (2013). HDL and endothelial protection. Br. J. Pharmacol..

[5] Cockerill G.W., Rye K.A., Gamble J.R., Vadas M.A., Barter P.J. (1995). High-density lipoproteins inhibit cytokine-induced expression of endothelial cell adhesion molecules. Arterioscler. Thromb. Vasc. Biol..

[6] Cockerill G.W., Huehns T.Y., Weerasinghe A., Stocker C., Lerch P.G., Miller N.E. (2001). Elevation of plasma high-density lipoprotein concentration reduces interleukin-1-induced expression of E-selectin in an in vivo model of acute inflammation. Circulation.

[7] Murphy A.J., Woollard K.J., Hoang A., Mukhamedova N., Stirzaker R.A., McCormick S.P.A. (2008). High-density lipoprotein reduces the human monocyte inflammatory response. Arterioscler. Thromb. Vasc. Biol..

[8] Bricarello D.A., Mills E.J., Petrlova J., Voss J.C., Parikh A.N. (2010). Ganglioside embedded in reconstituted lipoprotein binds cholera toxin with elevated affinity [S]. J. Lipid Res..

[9] Zhu B., Luo G., Feng Y., Yu M., Zhang J., Wei J. (2018). Apolipoprotein M protects against lipopolysaccharide-induced acute lung injury via sphingosine-1-phosphate signaling. Inflammation.

[10] Murphy A.J., Woollard K.J., Suhartoyo A., Stirzaker R.A., Shaw J., Sviridov D. (2011). Neutrophil activation is attenuated by high-density lipoprotein and apolipoprotein A-I in in vitro and in vivo models of inflammation. Arterioscler. Thromb. Vasc. Biol..

[11] Bao Dang Q., Lapergue B., Tran-Dinh A., Diallo D., Moreno J.-A., Mazighi M. (2013). High-density lipoproteins limit neutrophil-induced damage to the blood-brain barrier in vitro. J. Cereb. Blood Flow Metab..

[12] Ulevitch R.J., Johnston A.R., Weinstein D.B. (1979). New function for high density lipoproteins. Their participation in intravascular reactions of bacterial lipopolysaccharides. J. Clin. Invest..

[13] Levels J.H., Abraham P.R., van den Ende A., van Deventer S.J. (2001). Distribution and kinetics of lipoprotein-bound endotoxin. Infect. Immun..

[14] Levels J.H.M., Abraham P.R., Van Barreveld E.P., Meijers J.C.M., Van Deventer S.J.H. (2003). Distribution and kinetics of lipoprotein-bound lipoteichoic acid. Infect. Immun..

[15] Tanaka S., Labreuche J., Drumez E., Harrois A., Hamada S., Vigué B. (2017). Low HDL levels in sepsis versus trauma patients in intensive care unit. Ann. Intensive Care.

[16] Van Leeuwen H.J., Heezius E.C.J.M., Dallinga G.M., Van Strijp J.A.G., Verhoef J., Van Kessel K.P.M. (2003). Lipoprotein metabolism in patients with severe sepsis. Crit. Care Med..

[17] Cirstea M., Walley K.R., Russell J.A., Brunham L.R., Genga K.R., Boyd J.H. (2017). Decreased high-density lipoprotein cholesterol level is an early prognostic marker for organ dysfunction and death in patients with suspected sepsis. J. Crit. Care.

[18] Chien J.-Y., Jerng J.-S., Yu C.-J., Yang P.-C. (2005). Low serum level of high-density lipoprotein cholesterol is a poor prognostic factor for severe sepsis. Crit. Care Med..

[19] Grunfeld C., Pang M., Doerrler W., Shigenaga J.K., Jensen P., Feingold K.R. (1992). Lipids, lipoproteins, triglyceride clearance, and cytokines in human immunodeficiency virus infection and the acquired immunodeficiency syndrome. J. Clin. Endocrinol. Metab..

[20] Marin-Palma D., Sirois C.M., Urcuqui-Inchima S., Hernandez J.C. (2019). Inflammatory status and severity of disease in dengue patients are associated with lipoprotein alterations. PLoS One.

[21] Lighter J., Phillips M., Hochman S., Sterling S., Johnson D., Francois F. (2020). Obesity in patients younger than 60 Years is a risk factor for COVID-19 hospital admission. Clin. Infect. Dis..

[22] Wu Z., McGoogan J.M. (2020). Characteristics of and important lessons from the coronavirus disease 2019 (COVID-19) outbreak in China: summary of a report of 72 314 cases from the Chinese center for disease control and prevention. JAMA.

[23] Simonnet A., Chetboun M., Poissy J., Raverdy V., Noulette J., Duhamel A. (2020). High prevalence of obesity in severe acute respiratory syndrome coronavirus-2 (SARS-CoV-2) requiring invasive mechanical ventilation. Obesity.

[24] Richardson S., Hirsch J.S., Narasimhan M., Crawford J.M., McGinn T., Davidson K.W. (2020). Presenting characteristics, comorbidities, and outcomes among 5700 patients hospitalized with COVID-19 in the New York city area. JAMA.

[25] Wei C., Wan L., Yan Q., Wang X., Zhang J., Yang X. (2020). HDL-scavenger receptor B type 1 facilitates SARS-CoV-2 entry. Nat. Metab..

[26] Liu Y., Pan Y., Yin Y., Chen W., Li X. (2021). Association of dyslipidemia with the severity and mortality of coronavirus disease 2019 (COVID-19): a meta-analysis. Virol. J..

[27] Chidambaram V., Kumar A., Majella M.G., Seth B., Sivakumar R.K., Voruganti D. (2022). HDL cholesterol levels and susceptibility to COVID-19. eBioMedicine.

[28] Lassale C., Hamer M., Hernáez Á., Gale C.R., Batty G.D. (2021). Association of pre-pandemic high-density lipoprotein cholesterol with risk of COVID-19 hospitalisation and death: the UK Biobank cohort study. Prev. Med. Rep..

[29] Aparisi Á., Iglesias-Echeverría C., Ybarra-Falcón C., Cusácovich I., Uribarri A., García-Gómez M. (2021). Low-density lipoprotein cholesterol levels are associated with poor clinical outcomes in COVID-19. Nutr. Metab. Cardiovasc. Dis..

[30] Begue F., Tanaka S., Mouktadi Z., Rondeau P., Veeren B., Diotel N. (2021). Altered high-density lipoprotein composition and functions during severe COVID-19. Sci. Rep..

[31] Ding X., Zhang J., Liu L., Yuan X., Zang X., Lu F. (2020). High-density lipoprotein cholesterol as a factor affecting virus clearance in covid-19 patients. Respir. Med..

[32] Hu X., Chen D., Wu L., He G., Ye W. (2020). Declined serum high density lipoprotein cholesterol is associated with the severity of COVID-19 infection. Clinica Chim. Acta.

[33] Huang S., Zhou C., Yuan Z., Xiao H., Wu X. (2021). The clinical value of high-density lipoprotein in the evaluation of new coronavirus pneumonia. Adv. Clin. Exp. Med..

[34] Lv Z., Wang W., Qiao B., Cui X., Feng Y., Chen L. (2021). The prognostic value of general laboratory testing in patients with COVID-19. Clin. Lab. Anal..

[35] Masana L., Correig E., Ibarretxe D., Anoro E., Arroyo J.A., Jericó C. (2021). Low HDL and high triglycerides predict COVID-19 severity. Sci. Rep..

[36] Ouyang S.-M., Zhu H.-Q., Xie Y.-N., Zou Z.-S., Zuo H.-M., Rao Y.-W. (2020). Temporal changes in laboratory markers of survivors and non-survivors of adult inpatients with COVID-19. BMC Infect. Dis..

[37] Qin C., Minghan H., Ziwen Z., Yukun L. (2020). Alteration of lipid profile and value of lipids in the prediction of the length of hospital stay in COVID-19 pneumonia patients. Food Sci. Nutr..

[38] Sun J.T., Chen Z., Nie P., Ge H., Shen L., Yang F. (2020). Lipid profile features and their associations with disease severity and mortality in patients with COVID-19. Front. Cardiovasc. Med..

[39] Tanaka S., Tymowski C.D., Assadi M., Zappella N., Jean-Baptiste S., Robert T. (2020). Lipoprotein concentrations over time in the intensive care unit COVID-19 patients: results from the ApoCOVID study. PLoS One.

[40] Turgay Yıldırım Ö., Kaya Ş. (2021). The atherogenic index of plasma as a predictor of mortality in patients with COVID-19. Heart Lung.

[41] Wang Y., Lu X., Chen H., Chen T., Su N., Huang F. (2020). Clinical course and outcomes of 344 intensive care patients with COVID-19. Am. J. Respir. Crit. Care Med..

[42] Wang D., Li R., Wang J., Jiang Q., Gao C., Yang J. (2020). Correlation analysis between disease severity and clinical and biochemical characteristics of 143 cases of COVID-19 in Wuhan, China: a descriptive study. BMC Infect. Dis..

[43] Zhang B., Dong C., Li S., Song X., Wei W., Liu L. (2020). Triglyceride to high-density lipoprotein cholesterol ratio is an important determinant of cardiovascular risk and poor prognosis in coronavirus disease-19: a retrospective case series study. Diabetes Metab. Syndr. Obes..

[44] Zhang Q., Wei Y., Chen M., Wan Q., Chen X. (2020). Clinical analysis of risk factors for severe COVID-19 patients with type 2 diabetes. J. Diabetes Complications.

[45] Al-Zadjali J., Al-Lawati A., Al Riyami N., Al Farsi K., Al Jarradi N., Boudaka A. (2024). Reduced HDL-cholesterol in long COVID-19: a key metabolic risk factor tied to disease severity. Clinics.

[46] Deuel J.W., Lauria E., Lovey T., Zweifel S., Meier M.I., Züst R. (2022). Persistence, prevalence, and polymorphism of sequelae after COVID-19 in unvaccinated, young adults of the Swiss Armed Forces: a longitudinal, cohort study (LoCoMo). Lancet Infect. Dis..

[47] Li G., Du L., Cao X., Wei X., Jiang Y., Lin Y. (2021). Follow-up study on serum cholesterol profiles and potential sequelae in recovered COVID-19 patients. BMC Infect. Dis..

[48] Xu E., Xie Y., Al-Aly Z. (2023). Risks and burdens of incident dyslipidaemia in long COVID: a cohort study. Lancet Diabetes Endocrinol..

[49] Hilser J.R., Han Y., Biswas S., Gukasyan J., Cai Z., Zhu R. (2021). Association of serum HDL-cholesterol and apolipoprotein A1 levels with risk of severe SARS-CoV-2 infection. J. Lipid Res..

[50] Wang G., Zhang Q., Zhao X., Dong H., Wu C., Wu F. (2020). Low high-density lipoprotein level is correlated with the severity of COVID-19 patients: an observational study. Lipids Health Dis..

[51] Tanaka S., Couret D., Tran-Dinh A., Duranteau J., Montravers P., Schwendeman A. (2020). High-density lipoproteins during sepsis: from bench to bedside. Crit. Care.

[52] Chidambaram V., Shanmugavel Geetha H., Kumar A., Majella M.G., Sivakumar R.K., Voruganti D. (2022). Association of lipid levels with COVID-19 infection, disease severity and mortality: a systematic review and meta-analysis. Front. Cardiovasc. Med..

[53] McTaggart F., Jones P. (2008). Effects of statins on high-density lipoproteins: a potential contribution to cardiovascular benefit. Cardiovasc. Drugs Ther..

[54] Liao J.K., Laufs U. (2005). Pleiotropic effects of statins. Annu. Rev. Pharmacol. Toxicol..

[55] Blum A., Shamburek R. (2009). The pleiotropic effects of statins on endothelial function, vascular inflammation, immunomodulation and thrombogenesis. Atherosclerosis.

[56] Talasaz A.H., Sadeghipour P., Aghakouchakzadeh M., Dreyfus I., Kakavand H., Ariannejad H. (2021). Investigating lipid-modulating agents for prevention or treatment of COVID-19: JACC state-of-the-art review. J. Am. Coll. Cardiol..

[57] Talasaz A.H., Sadeghipour P., Bakhshandeh H., Sharif-Kashani B., Rashidi F., Beigmohammadi M.T. (2023). Atorvastatin versus placebo in ICU patients with COVID-19: ninety-day results of the INSPIRATION-S trial. Thromb. Haemost..

[58] Ghati N., Bhatnagar S., Mahendran M., Thakur A., Prasad K., Kumar D. (2022). Statin and aspirin as adjuvant therapy in hospitalised patients with SARS-CoV-2 infection: a randomised clinical trial (RESIST trial). BMC Infect. Dis..

[59] Florêncio de Mesquita C., Rivera A., Araújo B., Durães V.L., Queiroz I., Carvalho V.H. (2024). Adjunctive statin therapy in patients with covid-19: a systematic review and meta-analysis of randomized controlled trials. Am. J. Med..

[60] The REMAP-CAP Investigators (2023). Simvastatin in critically Ill patients with covid-19. N. Engl. J. Med..

[61] Xie Y., Xu E., Bowe B., Al-Aly Z. (2022). Long-term cardiovascular outcomes of COVID-19. Nat. Med..

[62] Chapman M.J., Goldstein S., Lagrange D., Laplaud P.M. (1981). A density gradient ultracentrifugal procedure for the isolation of the major lipoprotein classes from human serum. J. Lipid Res..

[63] Ballout R.A., Kong H., Sampson M., Otvos J.D., Cox A.L., Agbor-Enoh S. (2021). The NIH lipo-COVID study: a pilot NMR investigation of lipoprotein subfractions and other metabolites in patients with severe COVID-19. Biomedicines.

[64] Mietus-Snyder M., Suslovic W., Delaney M., Playford M.P., Ballout R.A., Barber J.R. (2022). Changes in HDL cholesterol, particles, and function associate with pediatric COVID-19 severity. Front. Cardiovasc. Med..

[65] Tanaka S., Begue F., Veeren B., Tran-Dinh A., Robert T., Tashk P. (2022). First recombinant high-density lipoprotein particles administration in a severe ICU COVID-19 patient, a multi-omics exploratory investigation. Biomedicines.

[66] Camont L., Chapman J., Kontush A. (2011). Functionality of HDL particles: heterogeneity and relationships to cardiovascular disease. Arch. Cardiovasc. Dis. Supplements.

[67] Begue F., Chemello K., Veeren B., Lortat-Jacob B., Tran-Dinh A., Zappella N. (2023). Plasma apolipoprotein concentrations are highly altered in severe intensive care unit COVID-19 patients: preliminary results from the LIPICOR cohort study. Int. J. Mol. Sci..

[68] Glomset J.A., Janssen E.T., Kennedy R., Dobbins J. (1966). Role of plasma lecithin:cholesterol acyltransferase in the metabolism of high density lipoproteins. J. Lipid Res..

[69] Stadler J.T., Mangge H., Rani A., Curcic P., Herrmann M., Prüller F. (2022). Low HDL cholesterol efflux capacity indicates a fatal course of COVID-19. Antioxidants.

[70] Papotti B., Macchi C., Favero C., Iodice S., Adorni M.P., Zimetti F. (2021). HDL in COVID-19 patients: evidence from an Italian cross-sectional study. J. Clin. Med..

[71] Souza Junior D.R., Silva A.R.M., Rosa-Fernandes L., Reis L.R., Alexandria G., Bhosale S.D. (2021). HDL proteome remodeling associates with COVID-19 severity. J. Clin. Lipidol..

[72] Grote K., Schaefer A.-C., Soufi M., Ruppert V., Linne U., Mukund Bhagwat A. (2024). Targeting the high-density lipoprotein proteome for the treatment of post-acute sequelae of SARS-CoV-2. Int. J. Mol. Sci..

[73] Kimhofer T., Lodge S., Whiley L., Gray N., Loo R.L., Lawler N.G. (2020). Integrative modeling of quantitative plasma lipoprotein, metabolic, and amino acid data reveals a multiorgan pathological signature of SARS-CoV-2 infection. J. Proteome Res..

[74] Stadler J.T., Habisch H., Prüller F., Mangge H., Bärnthaler T., Kargl J. (2023). HDL-related parameters and COVID-19 mortality: the importance of HDL function. Antioxidants.

[75] Marfia G., Navone S., Guarnaccia L., Campanella R., Mondoni M., Locatelli M. (2021). Decreased serum level of sphingosine-1-phosphate: a novel predictor of clinical severity in COVID-19. EMBO Mol. Med..

[76] Torretta E., Garziano M., Poliseno M., Capitanio D., Biasin M., Santantonio T.A. (2021). Severity of COVID-19 patients predicted by serum sphingolipids signature. Int. J. Mol. Sci..

[77] Lellouche F., Blais-Lecours P., Maltais F., Sarrazin J.-F., Rola P., Nguyen T. (2024). Ozanimod therapy in patients with COVID-19 requiring oxygen support. CHEST.

[78] Thomas M.J., Sorci-Thomas M.G. (2015). SAA: a link between cholesterol efflux capacity and inflammation?. J. Lipid Res..

[79] Vaisar T., Tang C., Babenko I., Hutchins P., Wimberger J., Suffredini A.F. (2015). Inflammatory remodeling of the HDL proteome impairs cholesterol efflux capacity. J. Lipid Res..

[80] Cho K.-H. (2021). Importance of apolipoprotein A-I and A-II composition in HDL and its potential for studying COVID-19 and SARS-CoV-2. Medicines.

[81] Ochoa-Ramírez L.A., De la Herrán Arita A.K., Sanchez-Zazueta J.G., Ríos-Burgueño E., Murillo-Llanes J., De Jesús-González L.A. (2024). Association between lipid profile and clinical outcomes in COVID-19 patients. Sci. Rep..

[82] Tanaka S., Genève C., Zappella N., Yong-Sang J., Planesse C., Louedec L. (2020). Reconstituted high-density lipoprotein therapy improves survival in mouse models of sepsis. Anesthesiology.

[83] Stasi A., Fiorentino M., Franzin R., Staffieri F., Carparelli S., Losapio R. (2023). Beneficial effects of recombinant CER-001 high-density lipoprotein infusion in sepsis: results from a bench to bedside translational research project. BMC Med..

[84] Cho K.-H., Kim J.-R., Lee I.-C., Kwon H.-J. (2021). Native high-density lipoproteins (HDL) with higher paraoxonase exerts a potent antiviral effect against SARS-CoV-2 (COVID-19), while glycated HDL lost the antiviral activity. Antioxidants.

[85] Faguer S., Del Bello A., Danet C., Renaudineau Y., Izopet J., Kamar N. (2022). Apolipoprotein-A-I for severe COVID-19-induced hyperinflammatory states: a prospective case study. Front. Pharmacol..

[86] Coelho D.R., Carneiro P.H., Mendes-Monteiro L., Conde J.N., Andrade I., Cao T. (2021). ApoA1 neutralizes proinflammatory effects of dengue virus NS1 protein and modulates viral immune evasion. J. Virol..

[87] Moreno J.-A., Ortega-Gomez A., Rubio-Navarro A., Louedec L., Ho-Tin-Noé B., Caligiuri G. (2014). High-density lipoproteins potentiate α1-antitrypsin therapy in elastase-induced pulmonary emphysema. Am. J. Respir. Cell Mol. Biol..

[88] Ding Y., Wang Y., Zhou J., Gu X., Wang W., Liu C. (2014). Direct cytosolic siRNA delivery by reconstituted high density lipoprotein for target-specific therapy of tumor angiogenesis. Biomaterials.

[89] Shahzad M.M.K., Mangala L.S., Han H.D., Lu C., Bottsford-Miller J., Nishimura M. (2011). Targeted delivery of small interfering RNA using reconstituted high-density lipoprotein nanoparticles. Neoplasia.

